# A human model of Buruli ulcer: The case for controlled human infection and considerations for selecting a *Mycobacterium ulcerans* challenge strain

**DOI:** 10.1371/journal.pntd.0011394

**Published:** 2023-06-29

**Authors:** Stephen Muhi, Joshua Osowicki, Daniel O’Brien, Paul D. R. Johnson, Sacha Pidot, Marcel Doerflinger, Julia L. Marshall, Marc Pellegrini, James McCarthy, Timothy P. Stinear

**Affiliations:** 1 Peter Doherty Institute for Infection and Immunity, The University of Melbourne, Melbourne, Victoria, Australia; 2 Victorian Infectious Diseases Service, Royal Melbourne Hospital, Parkville, Victoria, Australia; 3 Walter and Eliza Hall Institute of Medical Research, Parkville, Victoria, Australia; 4 Tropical Diseases Research Group, Murdoch Children’s Research Institute, The Royal Children’s Hospital, Parkville, Victoria, Australia; 5 Infectious Diseases Unit, Department of General Medicine, Royal Children’s Hospital Melbourne, Victoria, Australia; 6 Department of Paediatrics, University of Melbourne, Victoria, Australia; 7 Barwon Health, Geelong, Victoria, Australia; 8 Austin Health, Heidelberg, Victoria, Australia; Johns Hopkins University, UNITED STATES

## Abstract

Critical knowledge gaps regarding infection with *Mycobacterium ulcerans*, the cause of Buruli ulcer (BU), have impeded development of new therapeutic approaches and vaccines for prevention of this neglected tropical disease. Here, we review the current understanding of host–pathogen interactions and correlates of immune protection to explore the case for establishing a controlled human infection model of *M*. *ulcerans* infection. We also summarise the overarching safety considerations and present a rationale for selecting a suitable challenge strain.

## 1. Background

### 1.1. Disease burden

The neglected tropical disease, Buruli ulcer (BU), is a bacterial infection of skin and subcutaneous tissue. The causative bacteria *Mycobacterium ulcerans* was first described in contemporary literature by Australian researchers (MacCallum and colleagues) in 1948 [1]. Shortly thereafter, clinicians in African countries reported similar lesions, notably in the Buruli county (now Nakasongola District) of Uganda [2]. Cases of BU have since been identified globally, predominantly in West and Central Africa [3]. BU is the third most commonly reported mycobacterial infection after tuberculosis and leprosy, with an estimated 5,000 to 6,000 cases per year from 2004 to 2010 [4] although reducing in subsequent years [3]. In Africa, focal prevalence during outbreaks may reach as high as 15% [5].

In Victoria, Australia, where the climate is temperate, cases have continued to increase, with calls for an urgent scientific response [6]. Since first recognition of the “Bairnsdale ulcer” in the 1930’s [7], cases have been reported focally in regional, predominantly coastal areas, with an unpredictable pattern of geographical distribution. In early 2021, the epidemiology shifted unexpectedly, with an increase in incidence in the inner north-western suburbs of the Victorian state capital city Melbourne. Although investigations are ongoing, suggested explanations for this new urban focus have included increased rainfall due to multiple consecutive La Niña weather events, and the evolving complex relationship with native possums, which are known to be natural hosts [8] manifesting clinical disease and being geographically linked to human cases [9]. Like many neglected tropical diseases [10], the incidence of *M*. *ulcerans* is prone to shift in response to climate change and the increasingly complex relationship between humans and their environment.

### 1.2. Clinical disease

In Victoria, Australia, exposure is followed by a long incubation period, averaging 4 to 5 months [11,12]. *M*. *ulcerans* typically produces a localised skin and subcutaneous infection due to its growth requirement for cooler temperatures. Lesions often begin as a nodule, plaque or more rarely, oedema [13]. Ulcers typically develop slowly, and are characteristically painless, with an undermined edge, surrounding swelling and central necrosis. Left untreated, ulcers may either heal spontaneously, as reported in Australia [14], or progress in size and severity, leading to permanent contracture, deformity, and disability [15]. Contiguous infection of underlying bone has also been reported, principally in Africa [16]. Antibiotic treatment generally results in very high cure rates without the need for surgery, although treatment duration is long, and side-effects are common [17]. Although the disease is uncommonly lethal, vulnerable populations with inadequate access to healthcare are at risk of long-term disability due to advanced disease and scarring, particularly if antibiotic treatment (or surgery) is delayed.

### 1.3. Pathogenesis

The pathogenesis of BU is driven by production of the toxin mycolactone (ML), which has potent immunosuppressive properties [18]. Mycolactone acts by binding to the Sec61 translocon complex, preventing transport of newly synthesised proteins across the membrane of the endoplasmic reticulum [19]. By this mechanism, mycolactone irreversibly inhibits the production of numerous cytokines [20], including interleukin-2 (IL-2) [21,22] and interferon gamma (IFN-γ) [23]. The importance of IFN-γ is apparent in IFN-γ knockout mice, which have reduced capacity to kill intracellular bacilli [24] and faster disease progression [25]. Prolonged Sec61 inhibition also stimulates cytotoxic stress responses, with apoptosis [26] and dose-dependent necrosis [27], liberating bacilli [26,28] and inhibiting leukocyte infiltration [29] within the ulcer’s core. In the tissue surrounding this necrotic core, where mycolactone is less concentrated, a band of infiltrating leukocytes, comprising predominantly macrophages, contain intracellular bacilli [29,30]. This enables the centrifugal propagation of infection [31]. In mice, ML-negative isolates are avirulent, with a mononuclear infiltrate, intracellular bacilli, and granulomatous inflammation [29].

### 1.4. Human immune responses to *M*. *ulcerans*: Current knowledge

#### 1.4.1. Local cell-mediated immunity in humans

An understanding of the local and systemic cell-mediated immunity (CMI) to BU in humans, particularly during healing (following surgery, antibiotic therapy, or spontaneous healing) may uncover correlates of protection. Studies have consistently demonstrated that patients with BU have suppressed IFN-γ production during clinical disease, with relative suppression of Th1 responses, creating an environment where *M*. *ulcerans* evades mechanisms that usually control intracellular infection. As local immunity is likely to be more relevant than systemic immunity in this subcutaneous disease, intralesional immune profiles have been explored in human studies. Prévot and colleagues analysed the lesions of 14 patients in French Guiana, demonstrating high IL-10 but low IFN-γ mRNA levels in ulcers compared to high intralesional IFN-γ but low IL-10 mRNA levels in nodular lesions [32]. Kiszewski and colleagues analysed skin and subcutaneous tissue from 11 patients in Benin with BU, concluding that early ulcerative lesions had a predominantly immunosuppressive cytokine profile accompanied by high bacillary counts, whereas older lesions showed a mixed cytokine pattern, dominated by IFN-γ, together with low bacillary loads and granuloma formation [33]. Granuloma formation therefore likely represents a late event of the ulcerative stage, probably preceding healing. Schipper and colleagues studied 23 patients with active BU in Ghana, 7 with pre-ulcerative lesions and 16 with ulcerative disease, as well as 22 patients with healed BU [34]. They showed that patients with ulcerative and healed BUs produce significantly higher levels of IFN-γ compared to healthy controls, after *ex vivo* whole-blood stimulation with tuberculin purified protein derivative (PPD), and that patients with a granulomatous response produce higher IFN-γ levels than those without. As demonstrated by immunostaining of lesions at various stages of disease, further evidence supporting the protective effect of granulomatous inflammation in BU is the modest IFN-γ expression in the organising stage and marked IFN-γ expression during the granulomatous (healing) stage [34]. Schipper and colleagues were also the first to show that local IFN-γ production in granulomatous tissue was reflected by a systemic type 1 immune response to *M*. *ulcerans*, with a granulomatous response accompanied by high IFN-γ levels after whole-blood stimulation with tuberculin PPD [34].

#### 1.4.2. Systemic cell-mediated immunity in humans

There is inconsistency in the literature regarding the degree of systemic immunosuppression in BU. Gooding and colleagues analysed cellular immune responses in 14 patients from Victoria, Australia, 4 patients with active BU and 10 with healed lesions after surgical excision. They reported significantly reduced production of IFN-γ in response to stimulation with *M*. *ulcerans* whole cells compared to healthy controls [23]. Gooding and colleagues also studied 23 patients and 25 household contacts in far north Queensland (FNQ), finding that affected patients produced significantly greater Th2-type cytokines (IL-4, -5, -6, and -10) than unaffected household contacts [35]. Notably, only 1 patient had active disease, with the remainder having distant illness (mean of 9.4 years since illness) [35]. The only prospective analysis of the human immune response was a household contact participant of the larger trial in FNQ, who demonstrated a type 2 cytokine response 3 months after surgical excision of BU, failing to produce IFN-γ and IL-12, but with preserved IL-4 and IL-10 production upon peripheral blood mononuclear cell (PBMC) stimulation [36]. Prévot and colleagues also observed decreased IFN-γ production in stimulated PBMCs in patients with ulcerative lesions, compared to patients with nodular forms [32].

In a study performed in Ghana, Westenbrink and colleagues compared 23 patients with early-stage BU to 16 patients with late-stage BU. Using *in vitro* whole-blood stimulation with phytohemagglutinin (PHA) and tuberculin PPD, they reported that patients with early-stage BU produced significantly lower levels of IFN-γ compared to late-stage BU and control patients; systemic IL-10 and IL-4 levels did not differ between patients and controls in this study [37]. Schipper and colleagues reasoned that reduced systemic IFN-γ responses previously reported [23,32,35,36] may be because patients may have not had enough time to develop a type 1 immune response. This is unlikely to explain the observed differences for the studies performed by Gooding and colleagues, which predominantly included patients late in the course of their disease, while the duration of illness in the patients analysed by Prévot and colleagues was indeed shorter [32]. An alternative explanation is that patients in Australia and French Guiana may have sought treatment earlier or had improved access to healthcare than African patients. Studies suggest that systemic suppression of *M*. *ulcerans*-specific IFN-γ resolves 5 to 10 months after surgical excision of BU [38] and 1 to 2 months following curative antibiotic treatment [13].

In a subsequent study evaluating cytokine and chemokine responses to BU in Ghana, Phillips and colleagues not only found that PHA stimulation-induced production of IFN-γ in whole blood was reduced in patients compared to healthy controls, but also that Th2 and Th17 responses were down-regulated in patients with BU. This systemic immunosuppression was reported to resolve following treatment. They also demonstrated that patients with nodular lesions had suppressed circulating chemokines, and suppression of these chemokines persisted during the ulcerative stage of disease [22]. Phillips and colleagues also reported that serum IL-4 was significantly up-regulated in patients with BU during antibiotic treatment compared to healthy control participants, possibly explaining paradoxical reactions in patients during antibiotic therapy, due to increased B- and T-cell proliferation and Th-2 cell differentiation [22]. The inhibition of professional phagocyte function has also been reported in dendritic cells (DCs). Coutanceau and colleagues found that ML suppresses the capacity of DCs to prime cellular immune responses, which may help explain the inhibition of IFN-γ production by T-cells and the fact that once BU lesions are excised, there is an intact Th1 response to the organism [39].

#### 1.4.3. Humoral immunity

Like in most mycobacterial infections, humoral immunity seems to play a lesser role in controlling *M*. *ulcerans* infection. A number of studies have shown that patients with BU demonstrate an antibody response to infection [23,40]. Exposed controls can also have similar antibody responses to infected patients [35]. Antibodies targeting an immunodominant 18kDa heat-shock protein (Hsp18) without close orthologues in *M*. *bovis* or *M*. *tuberculosis* were frequently found in the sera of patients with BU (75%) and in healthy household contacts (38%) but rarely in controls from non-endemic regions [41]. Pidot and colleagues used comparative genomics to identify 45 potential *M*. *ulcerans-*specific proteins, of which they were able to express and purify 33 in *Escherichia coli* [42]. When sera from 30 patients with BU, 24 healthy controls from the same endemic region and 30 healthy controls from a non-endemic region in Benin were screened for *M*. *ulcerans*-specific antibodies to these proteins, 7 proteins (including PKS domains ER, AT propionate, and KR-A) showed a significant difference between patient and non-endemic controls. They also found that IgG responses of endemic controls were not significantly different to those of patients for any of the 7 proteins [42]. In the largest serosurvey to date, Hsp18 IgG titres in supposedly non-endemic areas showed comparable seropositivity rates to endemic areas (33% versus 31%, respectively) [43]. This suggests that unrecognised host factors may influence the development of clinical disease.

The production of ML-specific antibodies in laboratory animals has been challenging because of the molecule’s immunosuppressive nature and its small, lipid structure. Foulon and colleagues demonstrated the presence of neutralising antibodies in a mouse strain that displays spontaneous healing properties following infection with *M*. *ulcerans*. These neutralising antibodies were present in local tissue, but not in sera. Although ML is recognised by antibodies recovered from the lesions of 60% of patients from Benin with PCR-confirmed BU [44], the role of these antibodies in clinical disease remains unclear.

## 2. Role of a controlled human infection model for *M*. *ulcerans*

Due to the sporadic and highly focal nature of the epidemiology of BU, clinical trials testing vaccine efficacy among participants living in endemic areas are likely to be underpowered and expensive. A systematic review of BU vaccines [45] has illustrated the progress made towards vaccine development, with 28 studies describing vaccination approaches using various targets. The reviewers highlighted that, although several early stage candidate vaccines have emerged, other than *M*. *bovis* bacille Calmette–Guérin (BCG), none have progressed to efficacy testing [45]. More recent systems immunology approaches have resulted in the identification of additional vaccination candidates with attractive properties [46]. Thus, a number of promising candidates merit further investigation, but there is a lack of a path to efficacy testing.

Testing vaccines on healthy human volunteers, deliberately infected with *M*. *ulcerans* in a controlled fashion with a typical clinical phenotype, offers the potential for establishing safety and protective efficacy of a vaccine candidate much faster than would be possible using a randomised controlled trial in the field. A BU controlled human infection model (CHIM) could take a prominent position early in the development pipeline of vaccines or experimental therapies. CHIMs have been established to study a range of viral, bacterial, and parasitic pathogens. They are particularly valuable for complex and uncommon pathogens, allowing researchers to comprehensively interrogate the immunobiology of disease, while providing a biological signal of vaccine efficacy and identifying immunological correlates of protection, efficiently enabling subsequent stratification of vaccine development [47].

Vaccine efficacy can be studied in a CHIM of 10 to 100 participants, providing confidence to proceed to Phase 2 and 3 clinical trials where sample sizes of thousands of participants are typically required, with some confidence that the experimental intervention would be effective [48]. With a high (universal) infection rate in control participants, the number of participants required is small and readily defined [49]. For example, in a simple two-arm (placebo versus vaccine) trial with 90% power to detect a dichotomous outcome (lesion versus no lesion) at a *p* value of 0.05 and with vaccine efficacy of 70%, a CHIM with 100% attack rate would require 14 participants (7 participants per arm). If the attack rate was reduced to 80%, then a vaccine with 70% efficacy would require 18 participants per arm. In anticipation of dropouts, the sample size could be increased by 10% to 20% to compensate, without significantly affecting the economic feasibility of the study.

Beyond prophylactic interventions such as vaccines, other experimental therapeutic interventions may be tested using this model (Table 1). These interventions may be tested in parallel with vaccinations, as breakthrough infections may be diagnosed using experimental tests and treated using an experimental therapeutic product, with standard antibiotic therapy as a backup in case of treatment failure. Although a detailed review is beyond the scope of this paper, Roestenberg and colleagues have summarised the experimental infection of human volunteers and described the key ethical considerations, including novel models of neglected diseases [48].

**Table 1:** Questions that a BU CHIM may answer.

**1**	What is the natural history of natural infection?
**2**	What are the systemic and local immunological responses to low dose infection?
**3**	What are the immunological correlates of protection?
**4**	What proportion of people develop antibodies against *M*. *ulcerans*-specific antigens?
**5**	What kind of antibodies do people develop against *M*. *ulcerans*?
**6**	What is the immunological basis for paradoxical reactions?
**7**	Can paradoxical reactions be predicted based on systemic immunological parameters?
**8**	What are the immunological correlates of spontaneous healing?
**9**	Can *M*. *bovis* BCG protect people from low-dose *M*. *ulcerans* infection? [45]
**10**	Can candidate vaccines delay or prevent clinical disease?
**11**	Can clinical disease be prevented by using early, short courses of antibiotics prior to the onset of clinical disease? (i.e., chemoprophylaxis)
**12**	Can abbreviated antibiotic courses (e.g., 4 to 6 weeks of rifampicin and clarithromycin) be used to treat early disease?
**13**	Can ultra-short treatment with telacebac [52] successfully treat BU in humans?
**14**	Can *M*. *ulcerans-*targeted bacteriophages be used in the management of BU?
**15**	Can non-antibiotic options be useful in the treatment of early (pre-ulcerative or small) lesions? For example, local heat therapy (thermotherapy) [53]

BU, Buruli ulcer; CHIM, controlled human infection model.

There is no precedent for an *M*. *ulcerans* CHIM, with *M*. *bovis* BCG being the only mycobacteriosis to be studied in a CHIM setting [50]. Minassian and colleagues at the Jenner Institute (Oxford, United Kingdom) developed the model to test intradermal BCG vaccination as a surrogate for *M*. *tuberculosis* infection (because there is presently no safe tuberculosis CHIM available) [50]. They hypothesised that an effective vaccine against *M*. *tuberculosis* should reduce replication of *M*. *bovis* BCG, in their study population. In this model, intradermal BCG load was quantified using skin biopsy specimens by both quantitative polymerase chain reaction (qPCR) and culture using colony-forming units (CFUs); immunological correlates of protection were investigated by comparing pre-challenge immune profiles against *M*. *bovis* BCG load after challenge [50].

Because *M*. *ulcerans* infection is not restricted to humans, animal models may provide preclinical proof-of-concept, but the genetic and immunological differences make it difficult to extrapolate vaccine efficacy to humans [51]. In summary, although there are valuable clues in retrospective human studies, attempts to find immunological correlates of immune protection by retrospective analyses are hampered by long follow-up timeframes and confounders associated with observational methodology. Prospectively collected data that links clinical disease and immunological responses from baseline (pre-exposure) to infection and disease resolution, with or without exposure to a candidate vaccine or other intervention, has the potential to address numerous questions in BU research.

## 3. Approach to BU CHIM development

### 3.1. Safety considerations

Human models have been safely established for pathogens that can cause severe clinical syndromes, including *Streptococcus pneumoniae*, *Streptococcus pyogenes*, *Vibrio cholerae*, *Plasmodium falciparum*, dengue virus, and SARS-CoV-2 [47]. Compared to these pathogens, natural infections with *M*. *ulcerans* are slower, less severe, and relatively non-invasive, with low mortality, usually restricted to skin and subcutaneous tissue. Nevertheless, the safety of participants is paramount and residual risks to volunteers need to be minimised using a range of approaches, including (1) a cautious approach to strain selection and meticulous manufacturing processes in accordance with Good Manufacturing Practice (GMP) standards and requirements for regulatory approval; (2) selection of healthy adults, according to eligibility criteria approved by a human research ethics committee; (3) close clinical monitoring and support throughout the study; (4) prompt administration of curative antibiotic therapy; and (5) end-to-end oversight by a safety committee that includes investigators independent of the study team.

Initial studies to develop new CHIMs are generally restricted to healthy adult volunteers, following a thorough informed consent process [54]. For a BU CHIM, exclusion criteria would include a history of keloid formation and risk factors for delayed wound healing (e.g., smoking). BU severity and complications are particularly problematic in children [55] and the elderly [56], so young adult participants are preferred. They should also be screened for blood-borne viruses, primary and secondary immunodeficiencies, as well as comorbidities such as diabetes, which is a known risk factor for severe oedematous lesions and may impair wound healing [57]. Drug–drug interactions are a particular concern for both rifampicin and clarithromycin, so any concurrent medications should be carefully checked for interactions. A history of BCG vaccination and previous exposure to tuberculosis is also relevant due to potential baseline cross-protection.

A clinically relevant BU model would have a longer incubation period than for most CHIM studies, although the *Schistosoma mansoni* model is a good example of an established model with a long incubation period [58]. As in the schistosomiasis model, participants must be able to adhere to an outpatient follow-up schedule that could incorporate telehealth and in-person appointments as appropriate to facilitate prompt clinical evaluation, initiation of timely antibiotic treatment (or the intervention being tested), and wound care. Frequent follow-up will be needed until the lesion has fully healed, and until 12 months from treatment initiation, after which relapse or paradoxical reactions are uncommon [59,60]. In the absence of a lesion, follow-up beyond the longest reported incubation period is proposed (e.g., 12 months after challenge). Follow-up would be more frequent after any pre-ulcerative lesion is noted, with more extensive local and systemic sampling procedures. Transmission is thought to be from a puncture wound or mosquito bite, and human-to-human transmission is not thought to occur [61] but is nevertheless readily prevented by covering wounds.

In broad terms, a human model of infection can only be established for non-severe disease syndromes that are self-limited or reliably treated, with very rare acute or chronic complications [54]. Oral antibiotic therapy is now the standard of care for BU, with surgery reserved for very large ulcers (to achieve skin coverage) or unusual cases that respond incompletely to antibiotics [62]. Surgery may also be useful for those who cannot tolerate or decline antibiotic therapy or to shorten antibiotic treatment [62]. The WHO recommended antibiotic regimen is oral rifampicin (10 mg/kg once daily) and clarithromycin (7.5 mg/kg twice daily) for 8 weeks, following the results of a randomised trial that demonstrated all-oral therapy was non-inferior to injectable aminoglycoside antibiotic therapy and cured 96% of participants with early, limited BU [63]. Notably, the majority of participants with an unsuccessful outcome in this trial were lost to follow up or did not adhere to protocol-directed wound care. Of 146 participants, 9 (6%) prescribed oral combination antibiotic therapy experienced an adverse event, none serious. One participant prescribed rifampicin/clarithromycin experienced ototoxicity and 2 experienced non-severe QTc prolongation [63]. Retrospective observational Australian data suggests that antibiotic complications are not uncommon, particularly in those of advanced age [56] and renal impairment [17]. In retrospective Australian studies, treatment failure is rare (approximately 1%), and identified risk factors include patient weight >90 kg, male sex, and immunosuppression, highlighting the importance of careful participant selection [64].

Spontaneous healing without treatment has been reported in a small number of immunocompetent patients [14], although it is unclear how and why some patients mount sterilising immune responses and others do not. After antibiotic completion, early limited lesions typically heal after a median of 16 weeks (interquartile range 8 to 25 weeks) [63]. Approximately 7% of BU patients in Victoria’s Bellarine Peninsula develop an acute oedematous form of BU and may benefit from preemptive treatment with corticosteroids to minimise tissue destruction [65]. Paradoxical reactions may give the impression of clinical deterioration despite appropriate therapy. These reactions are observed after a median of 39 days [59] in approximately 20% of patients undergoing antibiotic treatment [56,59], occasionally requiring corticosteroids to blunt the exaggerated immunological response [66] and prolonged antibiotic treatment in selected cases [59]. These reactions are associated with high bacterial loads [67], emphasising the importance of careful definition of the challenge inoculum.

We envisage that in a BU human challenge trial, participants would begin antibiotic treatment shortly after a lesion develops. Although intervening this early in the disease process should preclude the need for surgical intervention, it is possible that the cosmetic result after complete excision of a small and early limited lesion might be preferred by some participants, may allow for a shortened duration of antibiotic therapy, and have the benefit of supplying tissue samples of significant research value. Participants would require more regular outpatient monitoring once treatment was started, for wound care and to observe any adverse effects from antibiotic therapy. Prompt initiation of treatment, prior to the development of significant soft tissue necrosis, would reduce the likelihood of secondary infection, while wound care also minimises environmental exposure [68].

### 3.2. Guiding criteria for *M*. *ulcerans* CHIM strain selection

With the overarching goals of promoting participant safety and clinical relevance of a BU CHIM, we propose the following considerations for selecting *M*. *ulcerans* challenge strains.

## Suggested characteristics of an ideal *M*. *ulcerans* challenge strain

### The ideal *Mycobacterium ulcerans* challenge strain

1. Is not associated with severe clinical disease, either in its individual provenance or in geographically associated cases.
2. Causes a typical infection phenotype.
3. Is amenable to a biologically plausible route of entry.
4. Is susceptible to clinically relevant antibiotics.
5. Can be cultured in a non-toxic, animal-free medium without genetic or chemical modification.
6. Can be accurately enumerated to ensure consistent challenge dosing.
7. Retains viability after cryopreservation.
8. Produces the key virulence factor after *in vitro* culture (i.e., mycolactone).
9. Maintains a conserved repertoire of genes encoding candidate vaccine antigens.
10. Remains genetically stable during manufacture and challenge.

1. *Is not epidemiologically associated with severe clinical disease (either in its individual provenance or in geographically associated cases)*

A suitable *M*. *ulcerans* challenge strain should not be associated with a severe clinical phenotype. For example, the Australian isolate JKD8049 has been previously studied in murine models of *M*. *ulcerans* infection [69,70]. JKD8049 was obtained from an adult male in 2004, during an outbreak in Point Lonsdale, Victoria. The presentation was of a characteristic painless ulcer on the posterior calf, which was first noticed incidentally by an allied health practitioner.

Although Australian isolates of *M*. *ulcerans* belong to the classical lineage that also causes BU in Africa, infections acquired in Australia demonstrate notably different clinical characteristics in observational studies and serve as a useful comparison [71]. In Point Lonsdale, Victoria, Australia, an outbreak began abruptly in 2002, with BU affecting 79 people (48 residents and 31 visitors) [72]. A clinical description of 180 cases across the entire Bellarine Peninsula (an area including Point Lonsdale) during this period noted that 95% of patients had a single lesion and the majority of patients had a nodule or ulcer [73]. Only 5% of patients presented with oedema, the majority of whom were over 60 years of age, similar to observed disease severity across Victoria [74]. One patient had septic bursitis, and another had BU osteomyelitis [73]; this contrasts with osteomyelitis rates of approximately 6% in Benin [16]. In Benin, osteomyelitis has been observed to occur some distance from active or apparently healed BU lesions and even in some patients without any history of BU skin lesion [16]. This phenomenon has rarely been reported in Australia [75]. Given that *M*. *ulcerans* grows optimally at 30° to 33°C, the subacute haematogenous spread suggested by these cases is surprising, although isolates from Africa are more thermotolerant than those from temperate regions [76].

2. *Reproduces a typical infection phenotype*

A successful CHIM should be clinically relevant, safely and reliably reproducing a clinical infection endpoint that faithfully resembles what is seen after natural exposure [49]. In Australia, the most common site of infection in humans is the lower limb, between the knee and ankle [77]. In an Australian setting, using an Australian isolate such as JKD8049, the posterior calf appeals as a site for inoculation, equidistant from the knee and ankle joints, and separated from the underlying bone by the large gastrocnemius muscle, reducing the (already low) risk of septic arthritis and osteomyelitis by contiguous spread, and contractures due to scarring. A lesion at this site is also easily amenable to surgical excision, if required. Observational studies also suggest that it is not a site with an increased likelihood of oedematous lesions such as those involving the hand, elbow, or ankle [57]. *M*. *bovis* BCG vaccination provides a useful reference point for the expected local superficial scar in a BU CHIM.

Unlike other pathogens tested in CHIMs, *M*. *ulcerans* is not restricted to humans, and has been identified in numerous zoonotic hosts. The selected challenge strain may therefore be characterised phenotypically in a murine model, in order to demonstrate predictable patterns of virulence and immunological responses to infection. At least in the case of JKD8049, remarkably low doses are known to establish infection in mice. For this isolate, the infectious dose to infect 50% of mice (ID50) is known to be approximately 2.6 CFU [69], although 10^4^ to 10^6^ CFU have been used in most vaccine/challenge murine studies, using a variety of challenge strains [45]. Such unrealistically high doses are likely to be far greater than what is required to establish natural infection and may overwhelm immune responses and underestimate the true efficacy of candidate vaccines. The CHIM should therefore use a realistically low dose of *M*. *ulcerans*. The initial study to establish a human challenge model must also incorporate a dose-finding design, as the infectious dose in humans is unknown.

In order to understand the typical disease phenotype, it is anticipated that the study end point will be reached at the onset of ulceration, or alternatively, when any pre-ulcerative lesion has been present for a significant period of time. If a cellulitic or oedematous presentation is encountered, the study end point will be reached, and treatment will need prompt initiation.

3. *Is amenable to a biologically plausible route of entry*

Studies have previously demonstrated that *M*. *ulcerans* isolate JKD8049 can cause infection after inoculation procedures mimicking the hypothesised mosquito bite route of entry, and also after subcutaneous injection, using realistically low doses in mice [69]. Studies have also shown that abrasions are not amenable to initiating clinical infection, at least in a guinea pig model [78]. The ID50 curve reported by Wallace and colleagues [69] demonstrates that, according to this model, the number of organisms required to establish infection in ≥90% of mice is approximately 20 CFU. Needlestick puncture has shown that a low-dose mouse tail infection was achieved in 21 of 24 (88%) of mice (average approximately 30 CFU) [69], while approximately 14 to 20 CFU resulted in infection in a mouse tail model, with at least 80% of mice infected [79]. Therefore, an initial target range of approximately 20 to 30 CFU may be considered, although minor deviations above this range are unlikely to have a clinically significant impact.

Given the success of previous experiments at successfully initiating infection using a low-dose mechanical model of infection, skin-puncturing microtrauma is proposed as the most biologically plausible method of inoculation for a BU CHIM. Specifically, a 25-gauge (or smaller) hypodermic needle, used to inject up to 0.1 mL of culture material subcutaneously, approximately 2 to 3 mm under the skin, is proposed. This depth approximates the length of a mosquito proboscis [80], which is postulated to be a possible route of infection in Australia [69,72,81].

4. *Is susceptible to clinically relevant antibiotics*

Although clarithromycin is the preferred companion drug to rifampicin, fluoroquinolones (such as ciprofloxacin or moxifloxacin) may be used in combination with rifampicin when clarithromycin is unavailable, contraindicated, or poorly tolerated [82]. Australian guidelines [82] recommend the use of clarithromycin alongside a fluoroquinolone when rifampicin cannot be used, based on effectiveness in mouse models [83]. Antibiotic resistance in Australian isolates has not been reported, unlike African isolates, where rifampicin resistance is described [84]. An ideal challenge strain would therefore be susceptible to rifampicin, clarithromycin, and fluoroquinolones *in vitro*, and a known allergy to these antibiotics would be an exclusion criterion. Although there were previously no defined clinical breakpoints for *in vitro* susceptibility testing, the Clinical and Laboratory Standards Institute (CSLI) guidelines offer suggested susceptibility ranges to interpret minimum inhibitory concentrations (MICs) for slow-growing non-tuberculous mycobacteria such as *M*. *ulcerans* [85]. Authorities suggest that laboratories should establish their own in-house validation for fastidious non-tuberculous mycobacterial species [85]. If externally validated, such methodology may standardise *M*. *ulcerans* MIC testing for future work.

5. *Can be cultured in a nontoxic and safe minimal media*, *with minimal chemical modification to enrich growth*

One of the major limitations in *M*. *ulcerans* research is the organism’s slow growth, due to a reported doubling time of approximately 48 h. This results in the requirement for long incubation periods, generally up to 12 weeks, within a relatively narrow temperature window. The long incubation period also increases the opportunity for contamination, reinforcing the importance of performing all experimental work within strictly sterile conditions. Although there are numerous *M*. *ulcerans* isolates available for consideration, geographically diverse isolates are remarkably conserved, with minimal genetic diversity [86]. Nevertheless, the growth characteristics of various isolates should be investigated; a faster time to culture positivity would be an attractive option, minimising the opportunity for contamination. However, an isolate with rapid growth characteristics is not necessarily a requirement for a candidate CHIM strain; rather, an isolate which reliably reproduces a typical clinical phenotype should be prioritised. The standard for *M*. *ulcerans* liquid culture, Middlebrook 7H9, requires the addition of albumin and catalase to enrich growth; both are generally derived from bovine sources. This introduces the small but not insignificant risk of bovine spongiform encephalopathy (BSE), particularly if the origin of the product is from an endemic region.

Sauton’s medium is an alternative liquid culture medium, which is free of animal products, and has an established history of use for the culture of *M*. *bovis* BCG [87]. It contains nontoxic ingredients and is pH neutral [88]. Previous studies have demonstrated that *M*. *bovis* BCG retains virulence properties when cultured in this medium compared to research media [87], while *M*. *ulcerans* reportedly also retains the ability to produce mycolactone in this medium [89]. Although often routinely added to reduce clumping, surfactants (e.g., polysorbate/Tween) are ideally avoided, aiming to minimise chemical modification, particularly considering the presence of hydrophobic lipid-rich structures, including mycolactone, in *M*. *ulcerans*. Other alternatives to explore may include replacing animal containing supplements with synthetic proteins or proteins of non-animal origin. Such additional nutritional supplementation is anticipated to enhance *M*. *ulcerans* growth; vegetable protein alternatives, which are free of animal protein and nongenetically modified, also have a history of use in a CHIM [49].

6. *Can be enumerated with accuracy to ensure precise challenge dosing*

**Fig 1:**
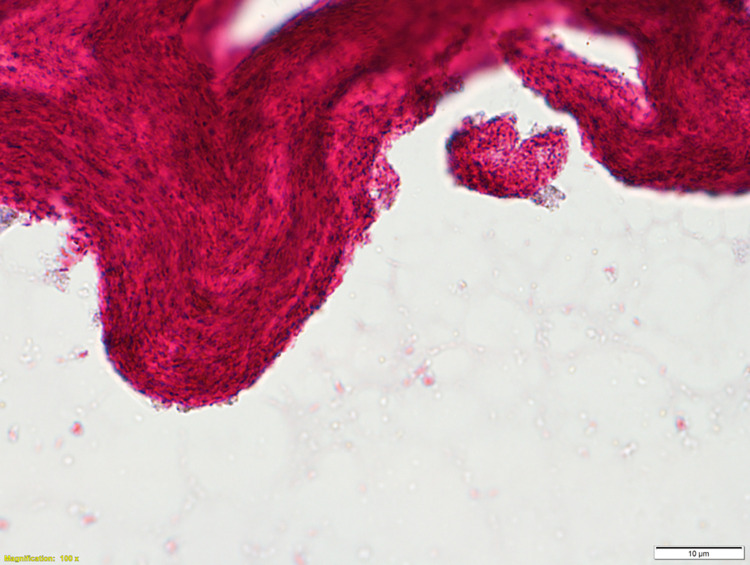
Ziehl–Neilson stain of *M*. *ulcerans* JKD8049 (×100 magnification with oil immersion) demonstrating clumping and cording characteristics, with innumerable acid-fast bacilli per clump when cultured in Sauton’s media without detergent.

An issue that is familiar to researchers of slow-growing mycobacteria, and particularly *M*. *ulcerans*, is the propensity to form clumps and biofilm, potentially containing hundreds or thousands of bacilli (Fig 1). *M*. *ulcerans* has a known dose-dependent relationship with clinical phenotype; mice receiving larger doses of *M*. *ulcerans* demonstrate earlier onset of ulceration and more rapid tissue loss [90]. Therefore, a single clump may significantly overdose challenge participants, considering the low doses proposed. In the absence of detergent, mechanical de-clumping methodologies will need to be explored.

7. *Remains viable after frozen storage*

Challenge doses of *M*. *ulcerans* created by any manufacturing process will require storage, generally by cryopreservation in a nontoxic substance (such as glycerol). Quality control processes of this sample will need to quantify its viability, including pre- and post-cryopreservation, and its stability after thawing using viable CFU count and/or quantitative PCR analysis.

8. *Produces the key virulence factor after in vitro culture (mycolactone)*

A clinically relevant BU model of infection would require that the selected isolate produces the main virulence toxin, mycolactone. Mycolactone production should be further characterised because minor sequence modifications in the ML genes of the pMUM plasmid (that encode the enzymes that synthesise the toxin) have produced a variety of ML congeners with variable virulence (A/B, C, D, E, and F). ML A/B (interconverting stereoisomers) produced by African strains are the most cytotoxic, while the potency is thought to be more attenuated in ML C (produced by Australian strains) and the other structural variants [91,92]. In murine fibroblast L929 cells treated with a series of synthetic mycolactones, ML F has been shown to be about 2 times less active and ML C about 15 times less active than ML A/B, respectively [93]. In addition to mycolactone C, Australian *M*. *ulcerans* strains also produce a fraction of ML A/B; Scherr and colleagues posit that the ML A/B portion may be more important for the pathogenesis caused by Australian strains than ML C [93]. In summary, the ability to produce mycolactone *in vitro* should be confirmed with liquid chromatography-mass spectroscopy.

9. *Maintains a conserved repertoire of genes encoding candidate vaccine antigens*

In order to test candidate vaccines against cell-surface antigens, whole-genome sequencing should confirm the presence of these genes in the challenge strain; these have been reviewed elsewhere [45]. Due to niche adaptation, global *M*. *ulcerans* isolates are remarkably conserved, so it is anticipated that most isolates should display a conserved repertoire of relevant antigens [94].

10. *Remains genetically stable during manufacture and challenge*

Serial whole-genome sequencing of the isolate, at multiple time points along the manufacturing cycle and following challenge, should (1) evaluate for the presence of spontaneous nucleotide polymorphisms; and (2) confirm the presence of an intact pMUM virulence plasmid.

## Conclusions

Our limited understanding of human BU immunology comes from small retrospective analyses. Prospective studies are needed to comprehensively interrogate correlates of protection. Although there are several challenges to establishing a safe and clinically relevant human infection model of BU, a human model offers the potential to revolutionise understanding of host–pathogen interactions and represents an appealing platform for evaluating vaccines and therapeutic interventions for this neglected disease. We have introduced the framework to guide the characterisation of candidate challenge strains and provided study design recommendations to carry towards first-in-human studies. Future work must examine the ethical framework for such a model and explore protocol considerations.

## References

[1] MacCallum P, Tolhurst JC, Buckle G, Sissons HA. A new mycobacterial infection in man. J Pathol Bacteriol. 1948;60(1):93–122. 18876541

[2] Clancey J, Dodge R, Lunn HF. Study of a mycobacterium causing skin ulceration in Uganda. Ann De La Société Belge De Médecine Tropicale. 1962;42:585–590. 14021473

[3] O’Brien DP, Jeanne I, Blasdell K, Avumegah M, Athan E. The changing epidemiology worldwide of *Mycobacterium ulcerans*. Epidemiol Infect. 2019;147:e19. 10.1017/S0950268818002662 PMC6518549 30293536

[4] Molyneux DH, Savioli L, Engels D. Neglected tropical diseases: progress towards addressing the chronic pandemic. Lancet. 2017;389(10066):312–325. doi: 10.1016/S0140-6736(16)30171-4 27639954

[5] Marston BJ, Diallo MO, Horsburgh CR, Diomande I, Saki MZ, Kanga JM, et al. Emergence of Buruli ulcer disease in the Daloa region of Cote D’Ivoire. Am J Trop Med Hyg. 1995;52(3):219–224. doi: 10.4269/ajtmh.1995.52.219 7694962

[6] O’Brien DP, Athan E, Blasdell K, Barro PD. Tackling the worsening epidemic of Buruli ulcer in Australia in an information void: time for an urgent scientific response. Med J Australia. 2018;208(7):287–289. doi: 10.5694/mja17.00879 29642808

[7] Alsop DG. The Bairnsdale ulcer. Aust NZ J Surg. 1972;41(4):317–319. 4554345

[8] Fyfe JAM, Lavender CJ, Handasyde KA, Legione AR, O’Brien CR, Stinear TP, et al. A major role for mammals in the ecology of *Mycobacterium ulcerans*. PLoS Neglect Trop Dis. 2010;4(8):e791. 10.1371/journal.pntd.0000791 PMC2919402 20706592

[9] Carson C, Lavender CJ, Handasyde KA, O’Brien CR, Hewitt N, Johnson PDR, et al. Potential wildlife sentinels for monitoring the endemic spread of human Buruli ulcer in south-east Australia. PLoS Neglect Trop Dis. 2014;8(1):e2668. doi: 10.1371/journal.pntd.0002668 24498452 PMC3907424

[10] Booth M. Climate change and the neglected tropical diseases. Adv Parasitol. 2018;100:39–126. doi: 10.1016/bs.apar.2018.02.001 29753342 PMC7103135

[11] Trubiano JA, Lavender CJ, Fyfe JAM, Bittmann S, Johnson PDR. The incubation period of Buruli ulcer *(Mycobacterium ulcerans infection)*. PLoS Neglect Trop Dis. 2013;7(10):e2463. 10.1371/journal.pntd.0002463 PMC3789762 24098820

[12] Loftus MJ, Trubiano JA, Tay EL, Lavender CJ, Globan M, Fyfe JAM, et al. The incubation period of Buruli ulcer (*Mycobacterium ulcerans* infection) in Victoria, Australia–Remains similar despite changing geographic distribution of disease. PLoS Neglect Trop Dis. 2018;12(3):e0006323. 10.1371/journal.pntd.0006323 PMC5875870 29554096

[13] Sarfo FS, Phillips RO, Ampadu E, Sarpong F, Adentwe E, Wansbrough-Jones M. Dynamics of the cytokine response to *Mycobacterium ulcerans* during antibiotic treatment for *M*. *ulcerans* disease (Buruli ulcer) in humans. Clin Vaccine Immunol. 2009;16(1):61–65. 10.1128/CVI.00235-08 PMC2620668 19005025

[14] O’Brien DP, Murrie A, Meggyesy P, Priestley J, Rajcoomar A, Athan E. Spontaneous healing of *Mycobacterium ulcerans* disease in Australian patients. Plos Neglect Trop Dis. 2019;13(2):e0007178. 10.1371/journal.pntd.0007178 PMC6396929 30779807

[15] Agbenorku P, Edusei A, Agbenorku M, Diby T, Nyador E, Nyamuame G, et al. Buruli ulcer induced disability in Ghana: A study at Apromase in the Ashanti region. Plastic Surg Int. 2012;2012:752749. 10.1155/2012/752749 PMC3362012 22666574

[16] Pommelet V, Vincent QB, Ardant MF, Adeye A, Tanase A, Tondeur L, et al. Findings in patients from Benin with osteomyelitis and polymerase chain reaction–confirmed *Mycobacterium ulcerans* infection. Clin Infect Dis. 2014;59(9):1256–1264. 25048846 10.1093/cid/ciu584

[17] O’Brien DP, Friedman D, Hughes A, Walton A, Athan E. Antibiotic complications during the treatment of *Mycobacterium ulcerans* disease in Australian patients. Intern Med J. 2017;47(9):1011–1019. 28585259 10.1111/imj.13511

[18] Baron L, Paatero AO, Morel JD, Impens F, Guenin-Macé L, Saint-Auret S, et al. Mycolactone subverts immunity by selectively blocking the Sec61 translocon. J Exp Med. 2016;213(13):2885–2896. doi: 10.1084/jem.20160662 27821549 PMC5154940

[19] Demangel C, High S. Sec61 blockade by mycolactone: A central mechanism in Buruli ulcer disease. Biol Cell. 2018;110(11):237–248. doi: 10.1111/boc.201800030 30055020

[20] Hall BS, Hill K, McKenna M, Ogbechi J, High S, Willis AE, et al. The pathogenic mechanism of the *Mycobacterium ulcerans* virulence factor, mycolactone, depends on blockade of protein translocation into the ER. PLoS Pathog. 2014;10(4):e1004061. 24699819 10.1371/journal.ppat.1004061 PMC3974873

[21] Pahlevan AA, Wright DJ, Andrews C, George KM, Small PL, Foxwell BM. The inhibitory action of Mycobacterium ulcerans soluble factor on monocyte/T cell cytokine production and NF-κB function. J Immunol. 1999;7(163):3928–3935. 10490994

[22] Phillips R, Sarfo FS, Guenin-Macé L, Decalf J, Wansbrough-Jones M, Albert ML, et al. Immunosuppressive signature of cutaneous *Mycobacterium ulcerans* infection in the peripheral blood of patients with Buruli ulcer disease. J Infect Dis. 2009;200(11):1675–1684. 19863437 10.1086/646615

[23] Gooding TM, Johnson PDR, Campbell DE, Hayman JA, Hartland EL, Kemp AS, et al. Immune response to infection with *Mycobacterium ulcerans*. Infect Immun. 2001;69(3):1704–1707. 11179346 10.1128/IAI.69.3.1704-1707.2001 PMC98075

[24] Bieri R, Bolz M, Ruf MT, Pluschke G. Interferon-γ Is a crucial activator of early host immune defense against *Mycobacterium ulcerans* infection in mice. Plos Neglect Trop Dis. 2016;10(2):e0004450. 10.1371/journal.pntd.0004450 PMC4749296 26863011

[25] Torrado E, Fraga AG, Logarinho E, Martins TG, Carmona JA, Gama JB, et al. IFN-γ–dependent activation of macrophages during experimental infections by *Mycobacterium ulcerans* is impaired by the toxin mycolactone. J Immunol. 2010;184(2):947–955. 20008288 10.4049/jimmunol.0902717

[26] Bieri R, Scherr N, Ruf MT, Dangy JP, Gersbach P, Gehringer M, et al. The macrolide toxin mycolactone promotes Bim-dependent apoptosis in Buruli ulcer through inhibition of mTOR. ACS Chem Biol. 2017;12(5):1297–1307. doi: 10.1021/acschembio.7b00053 28294596

[27] Adusumilli S, Mve-Obiang A, Sparer T, Meyers W, Hayman J, Small PLC. *Mycobacterium ulcerans* toxic macrolide, mycolactone modulates the host immune response and cellular location of M. ulcerans *in vitro* and *in vivo*. Cell Microbiol. 2005;7(9):1295–1304. 16098217 10.1111/j.1462-5822.2005.00557.x

[28] Ogbechi J, Hall BS, Sbarrato T, Taunton J, Willis AE, Wek RC, et al. Inhibition of Sec61-dependent translocation by mycolactone uncouples the integrated stress response from ER stress, driving cytotoxicity via translational activation of ATF4. Cell Death Dis. 2018;9(3):397. doi: 10.1038/s41419-018-0427-y 29540678 PMC5852046

[29] Oliveira MS, Fraga AG, Torrado E, Castro AG, Pereira JP, Filho AL, et al. Infection with *Mycobacterium ulcerans* induces persistent inflammatory responses in mice. Infect Immun. 2005;73(10):6299–6310. 16177301 10.1128/IAI.73.10.6299-6310.2005 PMC1230890

[30] Ruf MT, Steffen C, Bolz M, Schmid P, Pluschke G. Infiltrating leukocytes surround early Buruli ulcer lesions, but are unable to reach the mycolactone producing mycobacteria. Virulence. 2017;8(8):1918–1926. doi: 10.1080/21505594.2017.1370530 28873327 PMC5810495

[31] Torrado E, Fraga AG, Castro AG, Stragier P, Meyers WM, Portaels F, et al. Evidence for an intramacrophage growth phase of *Mycobacterium ulcerans*. Infect Immun. 2007;75(2):977–987. 17145944 10.1128/IAI.00889-06 PMC1828495

[32] Prévot G, Bourreau E, Pascalis H, Pradinaud R, Tanghe A, Huygen K, et al. Differential production of systemic and intralesional gamma interferon and interleukin-10 in nodular and ulcerative forms of Buruli disease. Infect Immun. 2004;72(2):958–965. doi: 10.1128/IAI.72.2.958-965.2004 14742541 PMC321599

[33] Kiszewski AE, Becerril E, Aguilar LD, Kader ITA, Myers W, Portaels F, et al. The local immune response in ulcerative lesions of Buruli disease. Clin Exp Immunol. 2006;143(3):445–451. doi: 10.1111/j.1365-2249.2006.03020.x 16487243 PMC1809619

[34] Schipper HS, Rutgers B, Huitema MG, Etuaful SN, Westenbrink BD, Limburg PC, et al. Systemic and local interferon-gamma production following *Mycobacterium ulcerans* infection. Clin Exp Immunol. 2007;150(3):451–459. 17900302 10.1111/j.1365-2249.2007.03506.x PMC2219368

[35] Gooding TM, Johnson PDR, Smith M, Kemp AS, Robins-Browne RM. Cytokine profiles of patients infected with *Mycobacterium ulcerans* and unaffected household contacts. Infect Immun. 2002;70(10):5562–5567. 12228283 10.1128/IAI.70.10.5562-5567.2002 PMC128325

[36] Gooding TM, Kemp AS, Robins-Browne RM, Smith M, Johnson PDR. Acquired T-helper 1 lymphocyte anergy following infection with *Mycobacterium ulcerans*. Clin Infect Dis. 2003;36:1076–1077. 12684923 10.1086/368315

[37] Westenbrink BD, Stienstra Y, Huitema MG, Thompson WA, Klutse EO, Ampadu EO, et al. Cytokine responses to stimulation of whole blood from patients with Buruli ulcer disease in Ghana. Clin Diagn Lab Immunol. 2005;12(1):125–129. doi: 10.1128/CDLI.12.1.125-129.2005 15642996 PMC540219

[38] Yeboah-Manu D, Peduzzi E, Mensah-Quainoo E, Asante-Poku A, Ofori-Adjei D, Pluschke G, et al. Systemic suppression of interferon-γ responses in Buruli ulcer patients resolves after surgical excision of the lesions caused by the extracellular pathogen *Mycobacterium ulcerans*. J Leukoc Biol. 2006;79(6):1150–1156. 16531561 10.1189/jlb.1005581

[39] Coutanceau E, Decalf J, Martino A, Babon A, Winter N, Cole ST, et al. Selective suppression of dendritic cell functions by *Mycobacterium ulcerans* toxin mycolactone. J Exp Med. 2007;204(6):1395–1403. 17517970 10.1084/jem.20070234 PMC2118602

[40] Dobos KM, Spotts EA, Marston BJ, Horsburgh CR, King CH. Serologic response to culture filtrate antigens of Mycobacterium ulcerans during Buruli ulcer disease. Emerg Infect Dis. 2000;6(2):158–164. doi: 10.3201/eid0602.000208 10756149 PMC2640848

[41] Diaz D, Döbeli H, Yeboah-Manu D, Mensah-Quainoo E, Friedlein A, Soder N, et al. Use of the immunodominant 18-kilodalton small heat shock protein as a serological marker for exposure to *Mycobacterium ulcerans*. Clin Vaccine Immunol. 2006;13(12):1314–1321. 17021247 10.1128/CVI.00254-06 PMC1694454

[42] Pidot SJ, Porter JL, Marsollier L, Chauty A, Migot-Nabias F, Badaut C, et al. Serological evaluation of *Mycobacterium ulcerans* antigens identified by comparative genomics. PLoS Neglect Trop Dis. 2010;4(11):e872. 10.1371/journal.pntd.0000872 PMC2970529 21072233

[43] Yeboah-Manu D, Röltgen K, Opare W, Asan-Ampah K, Quenin-Fosu K, Asante-Poku A, et al. Sero-epidemiology as a tool to screen populations for exposure to *Mycobacterium ulcerans*. PLoS Neglect Trop Dis. 2012;6(1):e1460. 10.1371/journal.pntd.0001460 PMC3254650 22253937

[44] Foulon M, Pouchin A, Manry J, Khater F, Robbe-Saule M, Durand A, et al. Skin-specific antibodies neutralizing mycolactone toxin during the spontaneous healing of *Mycobacterium ulcerans* infection. Sci Adv. 2020;6(9):eaax7781. 10.1126/sciadv.aax7781 PMC7043917 32133396

[45] Muhi S, Stinear TP. Systematic review of M. bovis BCG and other candidate vaccines for Buruli ulcer prophylaxis. Vaccine. 2021;39(50):7238–7252. doi: 10.1016/j.vaccine.2021.05.092 34119347

[46] Ishwarlall TZ, Adeleke VT, Maharaj L, Okpeku M, Adeniyi AA, Adeleke MA. Identification of potential candidate vaccines against *Mycobacterium ulcerans* based on the major facilitator superfamily transporter protein. Front Immunol. 2022;13:1023558. 36426350 10.3389/fimmu.2022.1023558 PMC9679648

[47] Choy RKM, Bourgeois AL, Ockenhouse CF, Walker RI, Sheets RL, Flores J. Controlled human infection models to accelerate vaccine development. Clin Microbiol Rev. 2022;35(3):e00008–e00021. doi: 10.1128/cmr.00008-21 35862754 PMC9491212

[48] Roestenberg M, Kamerling IMC, de Visser SJ. Controlled human infections as a tool to reduce uncertainty in clinical vaccine development. Front Med. 2018;5:297. doi: 10.3389/fmed.2018.00297 30420951 PMC6215823

[49] Osowicki J, Azzopardi KI, McIntyre L, Rivera-Hernandez T, Ong C, Baker C, et al. A controlled human infection model of group A streptococcus pharyngitis: Which strain and why? Msphere. 2019;4(1):e00647–e00618. doi: 10.1128/mSphere.00647-18 30760615 PMC6374595

[50] Minassian AM, Satti I, Poulton ID, Meyer J, Hill AVS, McShane H. A human challenge model for *Mycobacterium tuberculosis* using *Mycobacterium bovis* bacille Calmette-Guérin. J Infect Dis. 2012;205(7):1035–1042. 22396610 10.1093/infdis/jis012 PMC3295601

[51] Noll KE, Ferris MT, Heise MT. The Collaborative Cross: A systems genetics resource for studying host-pathogen interactions. Cell Host Microbe. 2019;25(4):484–498. doi: 10.1016/j.chom.2019.03.009 30974083 PMC6494101

[52] Almeida DV, Converse PJ, Omansen TF, Tyagi S, Tasneen R, Kim J, et al. Telacebec for ultrashort treatment of Buruli ulcer in a mouse model. Antimicrob Agents Chemother. 2020;64(6). doi: 10.1128/AAC.00259-20 32205344 PMC7269501

[53] Vogel M, Bayi PF, Ruf MT, Bratschi MW, Bolz M, Boock AU, et al. Local heat application for the treatment of Buruli ulcer: Results of a phase II open label single center non comparative clinical trial. Clin Infect Dis. 2016;62(3):342–350. doi: 10.1093/cid/civ883 26486698 PMC4706634

[54] Bambery B, Selgelid M, Weijer C, Savulescu J, Pollard AJ. Ethical criteria for human challenge studies in infectious diseases. Public Health Ethics. 2015;9(1):92–103. doi: 10.1093/phe/phv026 29731811 PMC5926904

[55] Walker G, Friedman DN, O’Brien MP, Cooper C, McDonald A, Callan P, et al. Paediatric Buruli ulcer in Australia. J Paediatr Child Health. 2020;56(4):636–641. doi: 10.1111/jpc.14704 31821679

[56] O’Brien DP, Friedman ND, Cowan R, Pollard J, McDonald A, Callan P, et al. *Mycobacterium ulcerans* in the elderly: more severe disease and suboptimal outcomes. PLoS Neglect Trop Dis. 2015;9(12):e0004253. 10.1371/journal.pntd.0004253 PMC4667883 26630648

[57] O’Brien DP, Friedman ND, McDonald A, Callan P, Hughes A, Athan E. Clinical features and risk factors of oedematous *Mycobacterium ulcerans* lesions in an Australian population: beware cellulitis in an endemic area. PLoS Neglect Trop Dis. 2014;8(1):e2612. 10.1371/journal.pntd.0002612 PMC3879256 24392172

[58] Langenberg MCC, Hoogerwerf MA, Koopman JPR, Janse JJ, Oosterhoud JK van, Feijt C, et al. A controlled human *Schistosoma mansoni* infection model to advance novel drugs, vaccines and diagnostics. Nat Med. 2020;26(3):326–32. 32066978 10.1038/s41591-020-0759-x

[59] O’Brien DP, Robson M, Friedman ND, Walton A, McDonald A, Callan P, et al. Incidence, clinical spectrum, diagnostic features, treatment and predictors of paradoxical reactions during antibiotic treatment of *Mycobacterium ulcerans* infections. BMC Infect Dis. 2013;13(1):416. 24007371 10.1186/1471-2334-13-416 PMC3854792

[60] Wynne JW, Stinear TP, Athan E, Michalski WP, O’Brien DP. Low incidence of recurrent Buruli ulcers in treated Australian patients living in an endemic region. PLoS Neglect Trop Dis. 2018;12(8):e0006724. 10.1371/journal.pntd.0006724 PMC6107289 30102695

[61] O’Brien DP, Wynne JW, Buultjens AH, Michalski WP, Stinear TP, Friedman ND, et al. Exposure risk for infection and lack of human-to-human transmission of *Mycobacterium ulcerans* disease. Australia Emerg Infect Dis. 2017;23(5):837–840. 28418294 10.3201/eid2305.160809 PMC5403060

[62] O’Brien DP, Callan P, Friedman ND, Athan E, Hughes A, McDonald A. *Mycobacterium ulcerans* disease management in Australian patients: the re-emergence of surgery as an important treatment modality. ANZ J Surg. 2019;89(6):653–658. 30239097 10.1111/ans.14829

[63] Phillips RO, Robert J, Abass KM, Thompson W, Sarfo FS, Wilson T, et al. Rifampicin and clarithromycin (extended release) versus rifampicin and streptomycin for limited Buruli ulcer lesions: a randomised, open-label, non-inferiority phase 3 trial. Lancet. 2020;395(10232):1259–1267. doi: 10.1016/S0140-6736(20)30047-7 32171422 PMC7181188

[64] O’Brien DP, Friedman ND, Walton A, Hughes A, Athan E. Risk factors associated with antibiotic treatment failure of Buruli ulcer. Antimicrob Agents Chemother. 2020;64(9). doi: 10.1128/AAC.00722-20 32571813 PMC7449191

[65] O’Brien DP, Huffam S. Pre-emptive steroids for a severe oedematous Buruli ulcer lesion: a case report. J Med Case Reports. 2015;9(1):98. doi: 10.1186/s13256-015-0584-x 25927351 PMC4428109

[66] Friedman ND, McDonald AH, Robson ME, O’Brien DP. Corticosteroid use for paradoxical reactions during antibiotic treatment for *Mycobacterium ulcerans*. PLoS Neglect Trop Dis. 2012;6(9):e1767. 10.1371/journal.pntd.0001767 PMC3459890 23029568

[67] Frimpong M, Agbavor B, Duah MS, Loglo A, Sarpong FN, Boakye-Appiah J, et al. Paradoxical reactions in Buruli ulcer after initiation of antibiotic therapy: Relationship to bacterial load. PLoS Neglect Trop Dis. 2019;13(8):e0007689. doi: 10.1371/journal.pntd.0007689 31449522 PMC6709892

[68] Pluschke G, Röltgen K, editors. Buruli ulcer, *Mycobacterium ulcerans* disease. 2019. 32091675

[69] Wallace JR, Mangas KM, Porter JL, Marcsisin R, Pidot SJ, Howden B, et al. *Mycobacterium ulcerans* low infectious dose and mechanical transmission support insect bites and puncturing injuries in the spread of Buruli ulcer. PLoS Neglect Trop Dis. 2017;11(4):e0005553. 10.1371/journal.pntd.0005553 PMC5406025 28410412

[70] Omansen TF, Porter JL, Johnson PDR, van der Werf TS, Stienstra Y, Stinear TP. In-vitro activity of avermectins against *Mycobacterium ulcerans*. PLoS Neglect Trop Dis. 2015;9(3):e0003549. 10.1371/journal.pntd.0003549 PMC4351077 25742173

[71] Käser M, Rondini S, Naegeli M, Stinear T, Portaels F, Certa U, et al. Evolution of two distinct phylogenetic lineages of the emerging human pathogen *Mycobacterium ulcerans*. BMC Evol Biol. 2007;7(1):177. 17900363 10.1186/1471-2148-7-177 PMC2098775

[72] Johnson PDR, Azuolas J, Lavender CJ, Wishart E, Stinear TP, Hayman JA, et al. *Mycobacterium ulcerans* in mosquitoes captured during outbreak of Buruli ulcer, southeastern Australia. Emerg Infect Dis. 2007;13(11):1653–1660. 18217547 10.3201/eid1311.061369 PMC3375796

[73] Boyd SC, Athan E, Friedman ND, Hughes A, Walton A, Callan P, et al. Epidemiology, clinical features and diagnosis of *Mycobacterium ulcerans* in an Australian population. Med J Australia. 2012;196(5):341–344. 22432674 10.5694/mja12.10087

[74] Tai AYC, Athan E, Friedman ND, Hughes A, Walton A, O’Brien DP. Increased severity and spread of *Mycobacterium ulcerans*, southeastern Australia. Emerg Infect Dis. 2018;24(1):58–64. 28980523 10.3201/eid2401.171070 PMC5749465

[75] Loftus MJ, Kettleton-Butler N, Wade D, Whitby RM, Johnson PD. A severe case of *Mycobacterium ulcerans* (Buruli ulcer) osteomyelitis requiring a below-knee amputation. Med J Australia. 2018;208(7):290–291. 29642809 10.5694/mja17.01158

[76] Eddyani M, Portaels F. Survival of *Mycobacterium ulcerans* at 37°C. Clin Microbiol Infect. 2007;13(10):1033–1035. 17697005 10.1111/j.1469-0691.2007.01791.x

[77] Yerramilli A, Tay EL, Stewardson AJ, Kelley PG, Bishop E, Jenkin GA, et al. The location of Australian Buruli ulcer lesions—Implications for unravelling disease transmission. PLoS Neglect Trop Dis. 2017;11(8):e0005800. doi: 10.1371/journal.pntd.0005800 28821017 PMC5584971

[78] Williamson HR, Mosi L, Donnell R, Aqqad M, Merritt RW, Small PLC. *Mycobacterium ulcerans* fails to infect through skin abrasions in a guinea pig infection model: Implications for transmission. PLoS Neglect Trop Dis. 2014;8(4):e2770. 10.1371/journal.pntd.0002770 PMC3983084 24722416

[79] Mangas KM, Buultjens AH, Porter JL, Baines SL, Marion E, Marsollier L, et al. Vaccine-specific immune responses against *Mycobacterium ulcerans* infection in a low-dose murine challenge model. Infect Immun. 2019;88(3). 10.1128/IAI.00753-19 PMC7035934 31818964

[80] Dixon AR, Vondra I. Biting innovations of mosquito-based biomaterials and medical devices. Dent Mater. 2022;15(13):4587. doi: 10.3390/ma15134587 35806714 PMC9267633

[81] Merritt RW, Walker ED, Small PLC, Wallace JR, Johnson PDR, Benbow ME, et al. Ecology and transmission of Buruli ulcer disease: A systematic review. PLoS Neglect Trop Dis. 2010;4(12):e911. doi: 10.1371/journal.pntd.0000911 21179505 PMC3001905

[82] O’Brien DP, Jenkin G, Buntine J, Steffen CM, McDonald A, Horne S, et al. Treatment and prevention of *Mycobacterium ulcerans* infection (Buruli ulcer) in Australia: guideline update. Med J Australia. 2014;200(5):267–270. 24641151 10.5694/mja13.11331

[83] Ji B, Chauffour A, Robert J, Lefrançois S, Jarlier V. Orally administered combined regimens for treatment of *Mycobacterium ulcerans* infection in mice. Antimicrob Agents Chemother. 2007;51(10):3737–3739. 17664316 10.1128/AAC.00730-07 PMC2043285

[84] Owusu E, Newman MJ, Kotey NK, Akumwena A, Bannerman E. Susceptibility profiles of *Mycobacterium ulcerans* isolates to streptomycin and rifampicin in two districts of the eastern region of Ghana. Int J Microbiol. 2016;2016:8304524. 28070190 10.1155/2016/8304524 PMC5192309

[85] Clinical and Laboratory Standards Institute. Susceptibility testing of Mycobacteria, Nocardiae, and other aerobic Actinomycetes; approved standard. Second edition. 2023. 31339680

[86] Röltgen K, Stinear TP, Pluschke G. The genome, evolution and diversity of *Mycobacterium ulcerans*. Infect Genet Evol. 2012;12(3):522–529. 22306192 10.1016/j.meegid.2012.01.018

[87] Venkataswamy MM, Goldberg MF, Baena A, Chan J, Jacobs WR, Porcelli SA. *In vitro* culture medium influences the vaccine efficacy of *Mycobacterium bovis* BCG. Vaccine. 2012;30(6):1038–1049. 22189700 10.1016/j.vaccine.2011.12.044 PMC3269512

[88] Parish T, Stoker NG, editors. Mycobacteria Protocols. 1998.

[89] Mve-Obiang A, Remacle J, Palomino JC, Houbion A, Portaels F. Growth and cytotoxic activity by *Mycobacterium ulcerans* in protein-free media. FEMS Microbiol Lett. 1999;181(1):153–157. 10564801 10.1111/j.1574-6968.1999.tb08838.x

[90] Bénard A, Sala C, Pluschke G. *Mycobacterium ulcerans* mouse model refinement for pre-clinical profiling of vaccine candidates. PLoS ONE. 2016;11(11):e0167059. 27893778 10.1371/journal.pone.0167059 PMC5125663

[91] Hong H, Stinear T, Porter J, Demangel C, Leadlay PF. A novel mycolactone toxin obtained by biosynthetic engineering. Chembiochem. 2007;8(17):2043–2047. doi: 10.1002/cbic.200700411 17907121 PMC2699038

[92] Mve-Obiang A, Lee RE, Portaels F, Small PLC. Heterogeneity of mycolactones produced by clinical isolates of *Mycobacterium ulcerans*: Implications for virulence. Infect Immun. 2003;71(2):774–783. 12540557 10.1128/IAI.71.2.774-783.2003 PMC145382

[93] Scherr N, Gersbach P, Dangy JP, Bomio C, Li J, Altmann KH, et al. Structure-activity relationship studies on the macrolide exotoxin mycolactone of *Mycobacterium ulcerans*. PLoS Neglect Trop Dis. 2013;7(3):e2143. 10.1371/journal.pntd.0002143 PMC3610637 23556027

[94] Stinear TP, Seemann T, Pidot S, Frigui W, Reysset G, Garnier T, et al. Reductive evolution and niche adaptation inferred from the genome of *Mycobacterium ulcerans*, the causative agent of Buruli ulcer. Genome Res. 2007;17(2):192–200. 17210928 10.1101/gr.5942807 PMC1781351

